# The dialogue between breast cancer and microorganisms

**DOI:** 10.3389/fcimb.2026.1738739

**Published:** 2026-05-28

**Authors:** Yan Zeng, Yuhang Jiang, Yinde Huang, Supeng Yin, Zeyu Yang, Fan Zhang

**Affiliations:** 1 Department of Breast and Thyroid Surgery, Chongging Academy of Medical Sciences, Chongqing General Hospital, School of Medicine, Chongqing University, Chongqing, China; 2 Department of Breast and Thyroid Surgery, Chongging Academy of Medical Sciences, Chongqing General Hospital, Chongqing, China; 3 Department of Laboratory Medicine, Chongqing University Jiangjin Hospital, Chongqing, China

**Keywords:** breast cancer, dysbiosis, estrogen, gut–mammary axis, immune microenvironment, metabolites, microbiota

## Abstract

Breast cancer is a complex pathological process involving multiple factors and stages, characterized by pronounced molecular and phenotypic heterogeneity. Its global incidence and mortality rates have shown a continuous upward trend. With the advancement of microbiome research, microbial communities have been recognized as key determinants influencing host health and disease states. Increasing evidence suggests a close association between breast tissue–resident and systemic microbiota and the initiation and progression of breast cancer. Specifically, microorganisms may be associated with abnormal proliferation and malignant transformation of mammary epithelial cells through diverse mechanisms, including the modulation of estrogen metabolism, production of bioactive metabolites, induction of chronic inflammation, and remodeling of the tumor microenvironment. In addition, certain microbes may directly interact with host cells, potentially inducing DNA damage and contributing to the transition from normal to malignant phenotypes. This review systematically summarizes the origins and compositional characteristics of the breast microbiota, with a particular focus on current evidence regarding its roles in breast cancer initiation, progression, metastasis, therapeutic response, and prognosis. Currently, the majority of evidence originates from cross-sectional studies and *in vitro*/*in vivo* model, to better evaluate the current evidence, the limitations of different research designs and the levels of evidence are summarized in **Table 1**, aiming to provide new theoretical insights and research perspectives for microbiota-based strategies in breast cancer diagnosis and therapy.

## 1 Introduction

**Figure 1:**
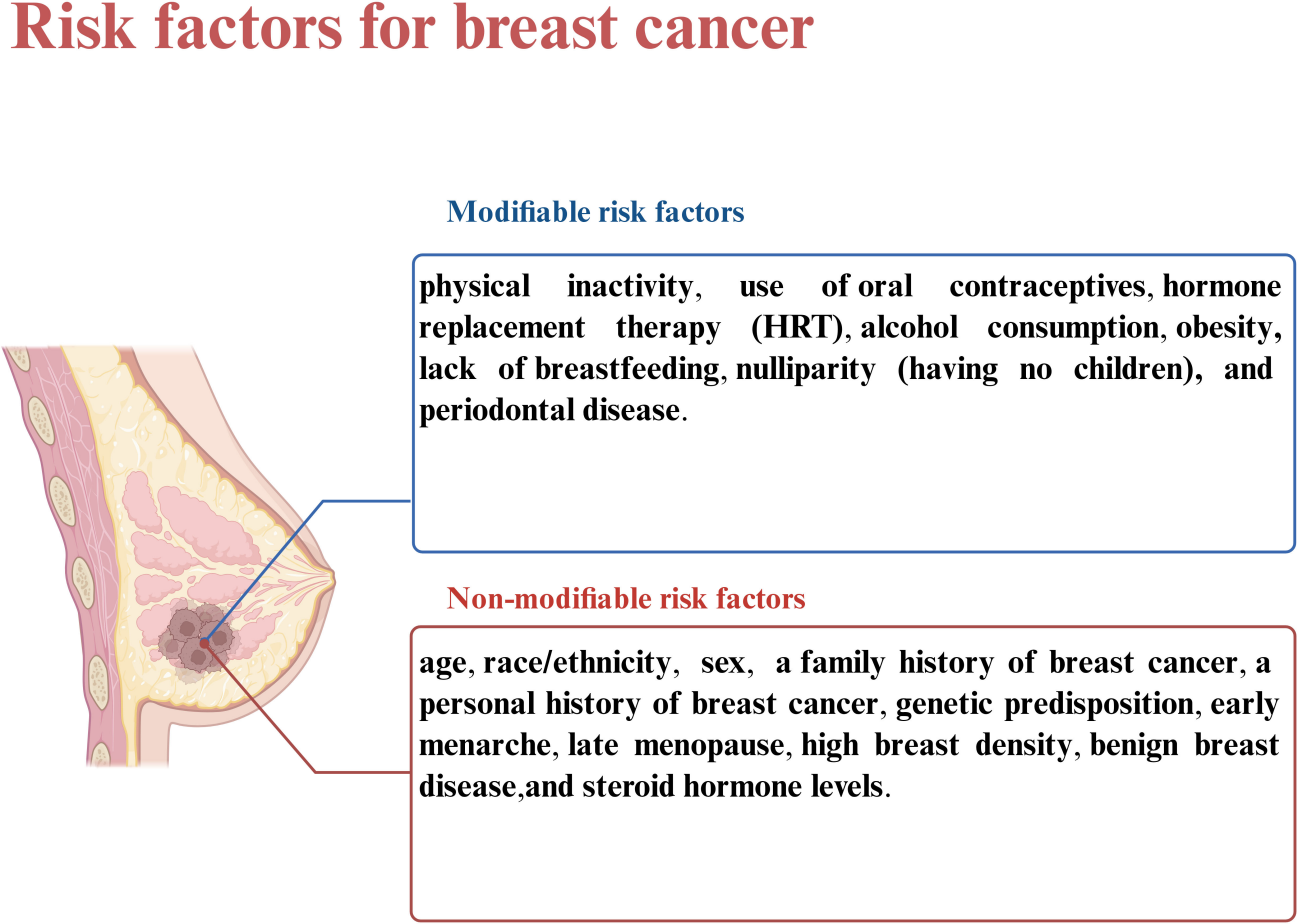
Risk factors for breast cancer. This includes both immutable factors and mutable factors. Created with Biorender.com.

Breast cancer (BC) is a common malignant tumor in women that originates from uncontrolled proliferation of mammary epithelial cells. In recent years, its global incidence has shown a persistent upward trend. According to the latest epidemiological projections, by 2040, the number of newly diagnosed breast cancer cases is expected to reach approximately 2,964,197 worldwide (Arnold et al., 2022). At present, breast cancer has become the second leading cause of cancer-related death among women globally (Giaquinto et al., 2024). This alarming situation is closely linked to its pronounced heterogeneity and complex pathogenesis. The development of breast cancer is influenced by a combination of factors. Non-modifiable risk factors include age, race, sex, family or personal history of breast cancer, genetic susceptibility, early menarche, delayed menopause, high breast density, history of benign breast disease, and elevated steroid hormone levels. Modifiable risk factors, on the other hand, encompass physical inactivity, use of oral contraceptives, hormone replacement therapy, alcohol consumption, obesity, breastfeeding practices, parity, and periodontal disease (Nandi et al., 2023) (**Figure 1**). Notably, approximately 70% of breast cancer cases cannot be fully explained by these established factors, suggesting substantial gaps in our current understanding of breast cancer etiology. Emerging evidence indicates that microorganisms—including bacteria and viruses—interact with various modifiable factors such as obesity, alcohol consumption, and periodontal disease, and may contribute to the initiation, progression, metastasis, and prognosis of breast cancer. These effects are potentially mediated through key mechanisms including regulation of hormone metabolism, induction of chronic inflammation, disruption of immune surveillance, remodeling of the tumor microenvironment, and metabolism of carcinogenic compounds.

**Table 1:** The limitations of different research designs and the levels of evidence.

Research field	Representative research	Evidence type	Model/Sample	Main findings	Limitations
Breast tissue microbiome composition	Urbaniak et al. (2016)	Cross-sectional cohort	Breast cancer vs. normal breast tissue (n=81)	Enrichmentof *Methylobacterium* and *Staphylococcus* in breast cancer tissues	Low-biomass samples are susceptible to contamination; limited resolution of 16S rRNA sequencing; correlational studies cannot establish causality
Gut microbiome and breast cancer	Thu et al. (2023)	Meta-analysis	32 studies	Gut microbiota in breast cancer patients exhibits specific compositional features	Can only establish correlation, not causation; limited by the quality of included original studies
Gut microbiome and breast cancer	Nejman et al. (2020)	Cross-sectional study	Breast, lung, pancreatic tumors and adjacent normal tissues (1,526 cases)	Breast tumors contain specific bacteria	Cannot determine causality
LCA and breast cancer	Mikó et al. (2018)	In vitro + In vivo	Breast cancer cell lines + mouse xenograft models	LCA inhibits breast cancer cell proliferation and migration	Not validated in humans
Butyric acid and breast cancer	Semaan et al. (2020)	In vitro	MCF-7 cells	Butyric acid induces G2/M phase arrest and apoptosis	Single cell line; lack of in vivo and human validation
*Fusobacterium nucleatum* and breast cancer metastasis	Parhi et al. (2020)	In vivo + clinical cohort	Mouse models + breast cancer tissues (approximately 200 cases)	*F. nucleatum* promotes DCIS progression and lung metastasis through MMP9	Differences exist between mouse models and human DCIS progression
Blood microbiome and breast cancer	Peters et al. (2024)	Case-control	Breast cancer patients vs. healthy control blood	Decreased α-diversity and enrichment of Ralstonia in breast cancer patients' blood	Low-biomass tissues are susceptible to contamination, leading to false positive results
Microbiome and treatment response	Di Modica et al. (2021)	In vivo + Clinical cohort	HER2+ breast cancer patients + in vivo	Butyrate enhances trastuzumab anti-tumor activity	Small sample size, not representative
Probiotics and immunotherapy	Álvarez-Mercado et al. (2023)	In vivo + Clinical observation	CTLA-4 inhibitor treatment models	Bifidobacterium enhances anti-PD-L1 therapeutic efficacy	Clinical data are correlational; strain specificity

Evidence level explanation: In vitro studies: Very low - Mechanistic exploration but lacks physiological complexity. In vivo models: Very low - Species differences limit translational applicability. Cross-sectional/case-control: Low - Correlational but cannot establish causality. Cohort studies: Moderate - Strong prospective design but numerous confounding factors. Meta-analysis: High - Limited by heterogeneity of original studies.

## 2 Potential origins of the breast tissue microbiota

For a long time, breast tissue was regarded as a sterile environment. However, recent studies have confirmed the presence of a rich and diverse microbial community within the mammary gland. The origins of these microorganisms remain a subject of ongoing debate, and several hypotheses have been proposed to explain their presence (Xu and Wang, 2025).

First, the gastrointestinal tract, which harbors the densest bacterial population in the human body, is considered a major potential source. Dysbiosis of the gut microbiota may impair intestinal barrier integrity, allowing bacteria or bacterial components to translocate into the bloodstream. Given the rich vascularization of breast tissue, these microorganisms have the potential to reach the mammary gland through hematogenous dissemination, leading to the proposal of a hypothetical “gut–breast axis” concept (Guo, 2024). Strong experimental evidence supports this hypothesis: following oral administration of *Lactobacillus strains*, identical bacteria have been detected in the mammary tissue of mice.

Second, the oral cavity, another important microbial reservoir, has also been implicated as a potential source of breast microbiota. Epidemiological studies have demonstrated a correlation between periodontitis and increased breast cancer risk, possibly mediated by hematogenous spread of oral pathogens to breast tissue (Zheng et al., 2022; Abolhassani et al., 2025). This finding further underscores the bloodstream as a key conduit for microbial migration to the mammary gland.

**Figure 2:**
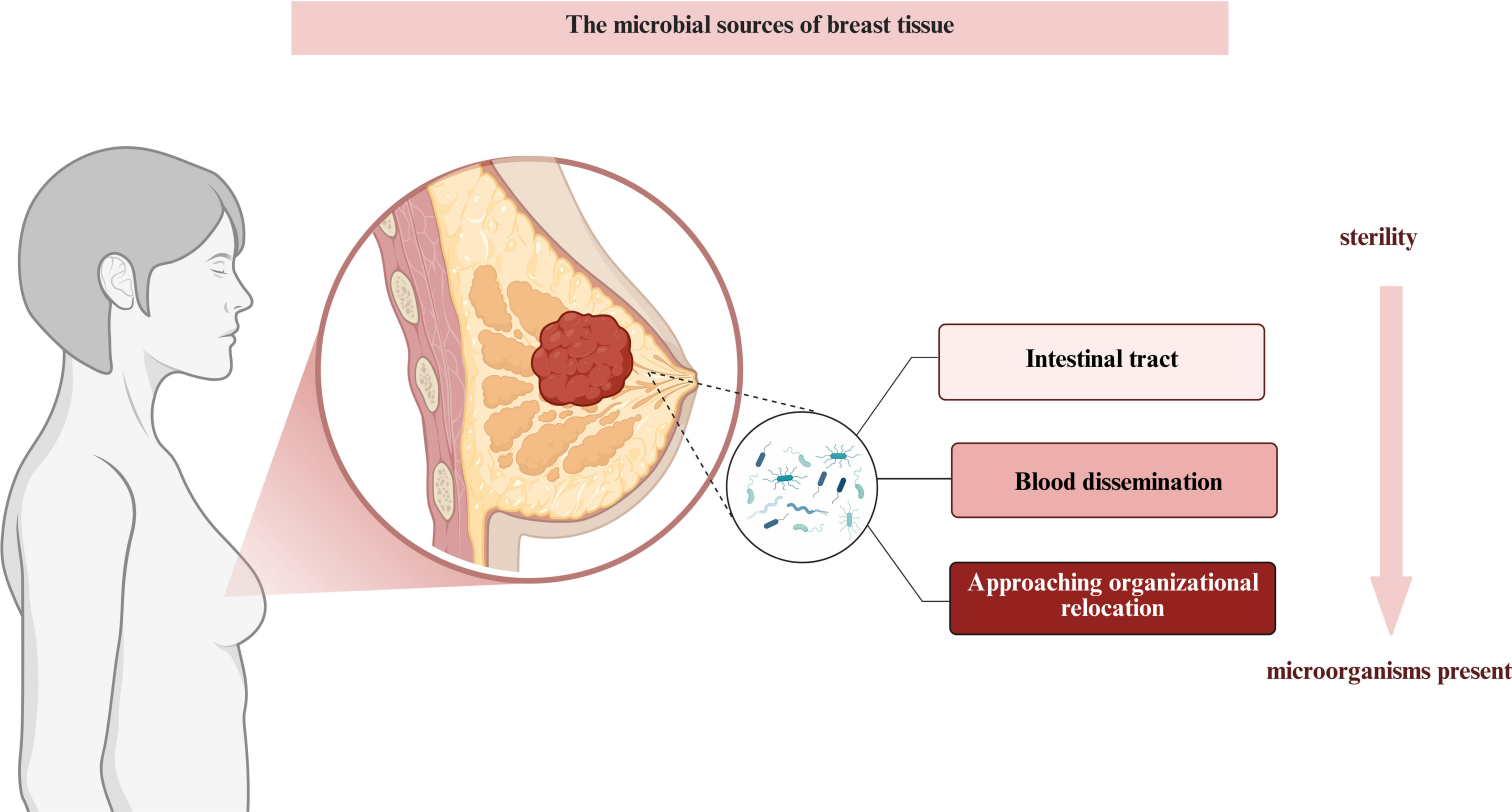
The origin of the breast microbiota is primarily explained by three hypotheses: 1) translocation from the gut (via the gut-mammary axis), 2) hematogenous spread through the bloodstream, and 3) migration from adjacent tissues. Created with Biorender.com.

Additionally, local translocation from adjacent tissues has been proposed as another source of breast-associated microbes. Nejman et al. reported that breast tumor tissues and their adjacent normal counterparts share a similar microbial composition, suggesting that microorganisms may colonize the breast via direct tissue contact or localized diffusion (Nejman et al., 2020) (**Figure 2**).

## 3 Breast tissue microbiota and breast cancer

Studies have shown that the microorganisms detected in breast tissue are not the result of external contamination but rather constitute a unique and stable microbial community. In patients with breast cancer, the composition of the breast microbiota undergoes marked alterations: *Methylobacterium radiotolerans* is significantly enriched, accompanied by increased abundance of *Enterobacteriaceae*, *Staphylococcus*, and *Bacillus*, while *Micrococcaceae* and *Sphingomonas* are notably reduced (Urbaniak et al., 2016; Hadzega et al., 2021). As tumor progression occurs, the microbial composition exhibits further dynamic shifts, characterized by a decrease in *Bacteroidetes* and an increase in *Agrococcus*, *Fusobacterium*, *Atopobium*, *Gluconacetobacter*, *Hydrogenophaga*, and *Lactobacillus*; this trend has also been validated using data from The Cancer Genome Atlas (TCGA) (Xuan et al., 2014; Bernardo et al., 2023a).

Moreover, the composition of the breast microbiota appears to differ across populations. In Canadian patients, *Bacillus* and *Acinetobacter* are found at higher levels; in Irish cohorts, *Enterobacteriaceae* and *Staphylococcus* predominate; whereas in Chinese breast cancer patients, significant enrichment of *Propionibacterium*, *Micrococcaceae*, *Caulobacteraceae*, *Rhodobacteraceae*, and *Methylobacteriaceae* has been observed. Among these, a decrease in *Bacteroidetes* and an increase in *Agrococcus* have been strongly associated with malignant transformation and tumor aggressiveness.

## 4 Gut microbiota and breast cancer

The breast is not an isolated microbial habitat; rather, the concept of a “gut–breast axis” has been proposed as a potential systemic regulatory pathway that has garnered increasing research interest in recent years. In healthy individuals, the gut microbiota is predominantly composed of members of the phyla *Firmicutes*, *Proteobacteria*, *Actinobacteria*, and *Bacteroidetes*, among which *Proteobacteria* represents the most abundant taxon (Thu et al., 2023).

Significant differences have been observed in both the composition and abundance of gut microbiota among individuals with normal breast tissue, benign breast lesions, and malignant tumors. Specifically, levels of *Porphyromonas* and *Alistipes* are elevated in breast cancer patients, whereas *Escherichia* and *Lactobacillus* are more enriched in those with benign lesions. Moreover, the overall microbial abundance and diversity are generally reduced in breast cancer patients, accompanied by a notable depletion of beneficial taxa such as *Odoribacter* sp., *Butyricimonas* sp., and *Coprococcus* sp., suggesting that gut microbiota dysbiosis may play a regulatory role in breast cancer pathogenesis (Bobin-Dubigeon et al., 2021; Peters et al., 2024).

Further analyses have revealed strong correlations between gut microbial composition and the clinicopathological features and tumor grades of breast cancer. For example, *Escherichia rectale*, *Mycobacterium smithii*, *Collinsella comes*, *Catenibacterium catus*, and *Collinsella aerofaciens* are more abundant in patients with lymph node–negative or low-grade breast cancer. In contrast, invasive breast cancer patients exhibit a higher relative abundance of Bacteroidetes but reduced levels of *Sphingomonadaceae* and *Ruminococcus*. In advanced-stage breast cancer, increased abundance of *Bacteroides*, *Clostridium coccoides*, *Clostridium leptum*, *Faecalibacterium*, and *Blautia* species has been documented (Nandi et al., 2023; Herrera-Quintana et al., 2024).

## 5 Blood microbiota and breast cancer

Beyond the gut microbiota, microorganisms and their components can also enter the systemic circulation, thereby exerting more direct regulatory effects on the breast tumor microenvironment. Recent studies have revealed distinctive alterations in the blood microbiota of breast cancer patients (Peters et al., 2024). Compared with healthy women, patients exhibit significantly reduced α-diversity in their circulating microbiota, characterized by enrichment of genera such as *Ralstonia*, *Methyloversatilis*, and *Campylobacter*, whereas *Aeribacillus*, *Thermincola*, and *Comamonas* are more prevalent in healthy individuals.

This controversy largely arises from the high susceptibility of blood-derived samples to environmental contamination and background bacterial signals introduced by reagents. In addition, current methodologies are unable to reliably discriminate between viable microorganisms, non-viable cells, and cell-free microbial DNA, thereby significantly undermining the accuracy and interpretability of detection results. As a result, the presence of circulating microbial DNA should not be interpreted as definitive evidence of a bona fide, viable microbial community in the bloodstream. Accordingly, conclusions regarding blood microbiota should be drawn with caution.

## 6 Microbial dysbiosis–induced alterations

Microbial dysbiosis is closely associated with the initiation and progression of breast cancer, and it is now widely recognized that multiple, interrelated mechanisms may collectively influence the malignant evolution of tumor cells. It can be mainly summarized into the following three aspects:

### 6.1 Hormone regulation

The gut microbiota plays a crucial role in maintaining host hormonal homeostasis. The risk of breast cancer progression is closely associated with circulating estrogen levels, particularly in hormone receptor–positive (HR+) subtypes, where estrogen binds to its receptors to initiate or enhance the transcription and expression of specific target genes, thereby promoting tumor cell proliferation. Estrogen signaling thus represents a central driver of tumorigenesis in this subtype (Hanker et al., 2020). The gut microbiota modulates estrogen metabolism primarily through the secretion of β-glucuronidase, an enzyme that catalyzes the deconjugation of estrogen metabolites (Heath et al., 2024).

Microorganisms such as *Clostridium, Ruminococcaceae*, *Escherichia coli*, and *Shigella* species play pivotal roles in this process by converting conjugated estrogens into their free, bioactive forms. Elevated abundance of these bacteria is significantly correlated with increased circulating free estrogen levels, thereby being associated with breast cancer progression (Fernández et al., 2018; Rea et al., 2018; Kwa et al., 2025). Evidence also indicates that elevated circulating estrogen in postmenopausal women constitutes an important risk factor for the development of estrogen receptor–positive breast cancer (Nandi et al., 2023). Furthermore, in HR+ breast cancer, disruption of the host–microbiota interplay and the consequent ecological imbalance can enhance the production of inflammation-related mediators associated with tumor progression, ultimately potentially contributing to cancer development.

### 6.2 Microbial metabolites and genotoxicity

The gut microbiota metabolizes dietary substrates such as fibers and proteins into a wide array of small-molecule metabolites that can enter the circulation and reach distant organs, including the breast, where they exert either pro-tumorigenic or anti-tumorigenic effects. Furthermore, certain bacterial species can directly synthesize genotoxic compounds that inflict DNA damage in host tissues.

#### 6.2.1 Genetic toxicity

Escherichia coli, through the production of colicins, can induce DNA double-strand breaks and genomic instability *in vitro*, thereby potentially increasing the carcinogenic potential of normal breast cells (Nejman et al., 2020). Additionally, enterotoxigenic Bacteroides fragilis (ETBF) secretes Bacteroides fragilis toxin (BFT), which not only stimulates proliferation of intraductal breast tumor cells but also enhances cancer cell self-renewal capacity through the β-catenin/Notch1 signaling pathway, thereby promoting tumor progression and metastasis (Parida et al., 2021). BFT can also induce NUMB protein phosphorylation, thereby increasing tumor stemness and chemoresistance (Chen G. et al., 2024; Ma et al., 2024).

#### 6.2.2 Microbial metabolites

(1) Bile acids: Among various metabolites, bile acids represent important bioactive molecules synthesized from cholesterol through complex enzymatic reactions by hepatocytes, playing a significant role in breast cancer pathogenesis. Lithocholic acid (LCA), a secondary bile acid predominantly produced by *Clostridium* species, exhibits a dual biological role: while it has been reported to possess carcinogenic properties in colorectal cancer, it exerts inhibitory effects in breast cancer through multiple mechanisms. On one hand, LCA activates the G protein–coupled bile acid receptor TGR5, suppress breast cancer cell proliferation and promote apoptosis (Luu et al., 2018). On the other hand, LCA downregulates the expression of nuclear factor erythroid 2–related factor 2 (NRF2) while upregulating Kelch-like ECH-associated protein 1 (KEAP1), disrupting the balance between pro-oxidant and antioxidant enzymes and ultimately impairing tumor cell proliferation.

Furthermore, LCA interferes with the epithelial–mesenchymal transition (EMT) process, enhances immune cell infiltration within the tumor microenvironment, and inhibits breast cancer progression by inducing *p53* expression and reducing vascular endothelial growth factor (VEGF) levels (Mikó et al., 2018; Kovács P. et al., 2019; Qi et al., 2022). Tumors with lower bile acid metabolic activity tend to display more aggressive phenotypes and harbor microbiota communities associated with malignancy (Režen et al., 2022). Decreased serum LCA levels observed in early-stage breast cancer patients suggest its potential as a biomarker for early diagnosis.

(2) Short-chain fatty acids (SCFAs):) As a significant category of metabolites generated through the fermentation of dietary polysaccharides by gut microbiota, these compounds exert multifaceted regulatory functions in the pathogenesis and progression of breast cancer. Common SCFAs include butyrate, propionate, and acetate. Studies have shown that sodium butyrate can induce apoptosis in colorectal cancer cells (HCT116) (Semaan et al., 2020); in breast cancer, its effects are more complex, involving mechanisms such as induction of G2/M cell cycle arrest, upregulation of caspase-10, and activation of intracellular calcium signaling, all of which promote tumor cell apoptosis (Wang Y. et al., 2016; Eslami-S et al., 2020; Jaye et al., 2022).

Moreover, sodium butyrate exhibits potent antitumor activity in HER2-positive breast cancer, functioning both as a monotherapy and in combination with trastuzumab to enhance anti-HER2 efficacy (Di Modica et al., 2021). In triple-negative breast cancer (TNBC), which lacks estrogen receptor, progesterone receptor, and HER2 expression and is characterized by high invasiveness and poor prognosis, conventional endocrine and targeted therapies are limited. Nevertheless, studies have demonstrated that sodium butyrate retains significant antitumor effects in TNBC models (Wang ZT. et al., 2016). Sodium propionate, a common food preservative, has also been shown to effectively inhibit breast cancer cell growth and induce apoptosis *in vitro* by mechanisms including blockade of the JAK2/STAT3 signaling pathway, cell cycle arrest, reactive oxygen species (ROS) generation, and p38 MAPK phosphorylation (Park et al., 2021).

(3)Other metabolites:Beyond SCFAs, other bacterial metabolites similarly contribute to breast cancer regulation. Cadaverine, produced via bacterial decarboxylation of lysine and arginine, can suppress breast cancer cell proliferation, migration, and invasion, and inhibit epithelial–mesenchymal transition (EMT); in 4T1 breast tumor models, cadaverine treatment markedly reduced tumor burden and metastasis (Kovács T. et al., 2019; Eslami-S et al., 2020; Guo et al., 2025). Indole derivatives represent another class of endogenous ligands for the aryl hydrocarbon receptor (AhR) that are produced through gut microbial metabolism. Experimental evidence demonstrates that indoles can inhibit the proliferation, stemness, and metastatic potential of breast cancer cells via AhR-mediated signaling pathways (Bernardo et al., 2023a). Sodium deoxycholate (DC), representing the predominant and stable physiological form of deoxycholic acid *in vivo*, demonstrates biphasic biological effects in mammary epithelial cells, with the underlying molecular mechanisms requiring further elucidation. Lactocellulose-derived peptides generated by *Lactobacillus casei* have been shown to exert potent cytotoxic effects against breast cancer cells. These effects are primarily mediated through modulation of transmembrane calcium ion flux and the induction of cell cycle arrest (Jaye et al., 2022). Furthermore, when combined with azithromycin, these peptides demonstrate a synergistic antitumor activity, suggesting potential for combinational therapeutic strategies (Nardo et al., 2024). As a key member of the gut-resident microbiota, *Clostridium* species can metabolically produce trimethylamine N-oxide (TMAO). This metabolite has been implicated in facilitating the maturation and activation of effector CD8^+^ T cells, thereby amplifying their antitumor immune functions. Notably, this immunomodulatory effect has been associated with pronounced tumor-suppressive activity in HER2/neu^+^ breast cancer models (Mirji et al., 2022; Wang et al., 2022; Lu et al., 2024). In addition, a range of gut microbiota–derived metabolites, including lignans, enterolignans, and colicins, have been reported to exhibit inhibitory potential against breast cancer progression (Vétizou et al., 2015; Di et al., 2018; Druzhinin et al., 2018; Mali et al., 2018; Álvarez-Mercado et al., 2023). In summary, microbial metabolites can exert both tumor-promoting and tumor-suppressive effects through a range of distinct and context-dependent mechanisms.

### 6.3 Immune regulation and inflammation

The microbial community plays a multifaceted role in the initiation and progression of breast cancer, primarily through its impact on immune regulation and inflammatory processes within the tumor microenvironment. For example, lipoteichoic acid (LTA), a structural component of *Staphylococcus aureus*, can activate the TLR2/NF-κB signaling pathway, leading to increased expression of pro-inflammatory cytokines such as IL-6 and TNF-α (Schauber et al., 2007; Zhao et al., 2008; Whelehan et al., 2011). This inflammatory cascade contributes to the formation of an immunosuppressive tumor microenvironment and may directly facilitate breast tumor growth (Ran et al., 2025). *Enterotoxigenic Bacteroides fragilis* (ETBF) not only contributes to tumorigenesis through genotoxic activity but also promotes the production of pro-inflammatory and tumor-associated cytokines, including IL-17A and IL-6. This, in turn, drives systemic inflammatory responses and reshapes the tumor microenvironment (Parida et al., 2023). *Staphylococcus epidermidis* has been reported to trigger localized inflammatory reactions, enhance regulatory T cell infiltration, and activate complement signaling, while also facilitating the polarization of macrophages toward a pro-tumorigenic M2 phenotype *in vitro*. By contrast, *Micrococcus luteus* has demonstrated tumor-suppressive potential *in vivo* by inhibiting mammary tumor growth and promoting the polarization of macrophages toward an anti-tumor M1 phenotype *in vitro* (Bernardo et al., 2023b). Several probiotic strains have also been implicated in protective effects against breast cancer. *Bifidobacterium* species can promote the differentiation of naïve T cells into regulatory T cells and enhance IL-10 secretion, thereby attenuating tumor progression through the modulation of local inflammatory responses and immune regulation. In contrast, *Lactobacillus acidophilus* is reported to drive a Th1-skewed immune profile, characterized by increased IL-12 and IFN-γ production alongside decreased IL-4 and TGF-β levels, which collectively support an antitumor immune environment. Similarly, *Lactobacillus casei* has been shown to inhibit breast cancer growth and metastasis by limiting macrophage infiltration within tumors while enhancing CD4^+^ and CD8^+^ T cell–mediated immune activity (Nandi et al., 2023). Moreover, dominant bacterial phyla such as Proteobacteria and Firmicutes may exert systemic immunomodulatory effects through the regulation of microbial metabolites, thereby influencing lymphocyte proliferation, chronic inflammatory status, and estrogen metabolism pathways, ultimately contributing to immune surveillance and tumor suppression in breast cancer (O’Connor et al., 2018).

Obesity has been established as a confirmed risk factor for triple-negative breast cancer (TNBC) (Pierobon and Frankenfeld, 2013). Excessive adipose tissue accumulation *in vivo* triggers tumor microenvironment remodeling, promoting breast cancer progression and metastasis processes, while exhibiting strong correlations with patient survival duration and overall survival rates (Papakonstantinou et al., 2022). High-fat dietary intervention can further restructure gut microbiota composition, through microbially-mediated leucine metabolism and polymorphonuclear myeloid-derived suppressor cell (PMN-MDSC) differentiation, thereby advancing tumor progression and compromising doxorubicin therapeutic responsiveness (Chen J. et al., 2024). Additional research demonstrates that in TNBC mouse models, doxorubicin treatment significantly alters gut microbial composition, leading to increased abundance of Akkermansia muciniphila (Bawaneh et al., 2022).

### 6.4 Distinct roles of different bacterial species in breast cancer

The microbial community represents a complex and dynamically balanced ecosystem. Rather than acting in isolation, the mechanisms described above interact in a coordinated manner to influence breast cancer progression. Distinct bacterial species contribute to breast cancer initiation and development through diverse and context-dependent pathways. A summary of representative bacterial taxa and their associated mechanisms is provided in **Table 2**.

**Table 2:** Main mechanisms of representative bacteria in breast cancer.

Bacteria	Primary mechanisms of action	Functional frameworks	Tumor effects
*Escherichia coli*	production of colibactin (causing DNA damage) (Urbaniak et al., 2016), LPS-induced epithelial-mesenchymal transition (EMT) that enhances migration and invasion (Hsu et al., 2011; Liu et al., 2021; Afroz et al., 2022), and secreted extracellular factors that stimulate angiogenesis (Bernardo et al., 2023a).	Metabolic/genotoxicity; immune regulation	Pro-tumorigenic
*Staphylococcus aureus*	LTA→TLR2/NF-κB→IL-6,TNF,shaping an immunosuppressive microenvironment (Schauber et al., 2007; Zhao et al., 2008; Whelehan et al., 2011; Ran et al., 2025)	immune regulation	Pro-tumorigenic
*Staphylococcus, Lactobacillus, and Streptococcus*	Modulating stress responses and impacting cancer cell viability, altering cytoskeletal architecture, and facilitating metastatic processes (Fu et al., 2022)	immune regulation	Pro-tumorigenic
*Fusobacterium nucleatum*	Inducing MMP-9 expression and basement membrane degradation; Fap2-Gal-GalNAc→TLR4 activation (Parhi et al., 2020; Li et al., 2023)	Immunity/Inflammation	Pro-tumorigenic
*Bacteroides fragilis* (ETBF)	BFT toxin activates Notch1/β-catenin signaling; induces IL-17A/IL-6; enhances stemness and chemoresistance (Parida et al., 2021; Parida et al., 2023; Chen G. et al., 2024; Ma et al., 2024)	Genotoxicity	Pro-tumorigenic
*Bacillus cereus*	Progesterone metabolism to 5αP (Bernardo et al., 2023a)	Metabolism / Hormone	Pro-tumorigenic
*Clostridium*	TMAO production activates CD8+ T cells (Mirji et al., 2022; Wang et al., 2022; Lu et al., 2024)	Immunoregulation and microbial metabolites	Anti-tumorigenic
*Corpus luteum micrococcus*	promote M1 phenotype	immune regulation	Anti-tumorigenic
*Staphylococcus epidermidis*	increase Treg infiltration, enhance M2 macrophage phenotype (Bernardo et al., 2023b)	immune regulation	Pro-tumorigenic
*Lactobacillus casei*	enhances CD4+ and CD8+ immune response (Nandi et al., 2023)	immune regulation	Anti-tumorigenic
Proteobacteria, Firmicutes	Regulating bacterial metabolites to affect lymphocyte proliferation, chronic inflammation, and estrogen metabolism to modulate the immune system, etc (O’Connor et al., 2018)	Hormone, Metabolism, Immunity	Anti-tumorigenic

## 7 Mechanisms linking microbiota to the transition from DCIS to IDC

Breast cancer development is a multistage, progressive pathological process, typically involving a sequential transformation of normal mammary ductal epithelium through hyperplasia, atypical hyperplasia, and ductal carcinoma *in situ* (DCIS), ultimately progressing to invasive ductal carcinoma (IDC) with metastatic potential. Among these stages, DCIS is considered the primary precancerous lesion of IDC, accounting for approximately 20%–30% of all breast cancer diagnoses.

A key clinical challenge lies in the fact that not all DCIS cases will progress to invasive malignancy. Studies indicate that only about 20%–40% of untreated DCIS cases ultimately evolve into IDC (Song et al., 2022). Currently, clinical risk assessment primarily relies on limited indicators such as histological grading, highlighting the urgent need for more precise biomarkers to identify DCIS subtypes at high risk of progression.

**Figure 3:**
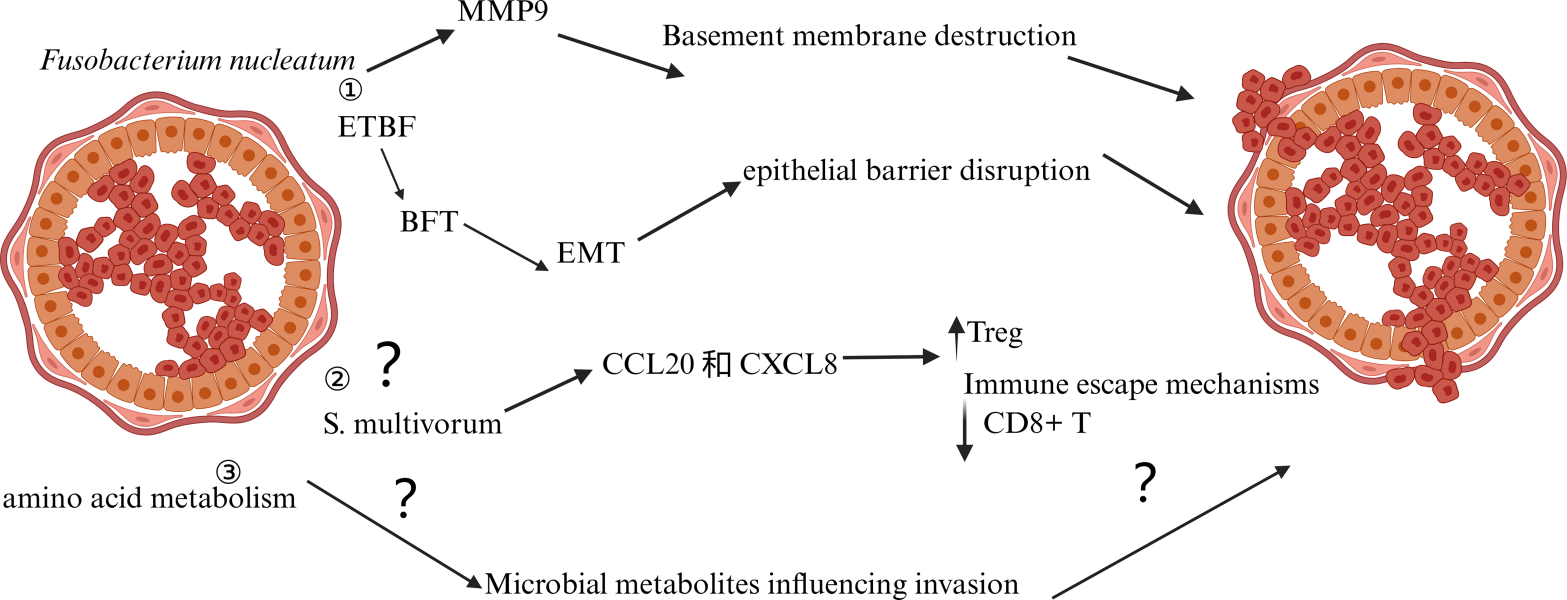
Potential mechanisms of microbe-promoted progression from DCIS to IDC. 1) potential main roles including disruption of epithelial barrier and basement membrane, 2)promotion of immune escape, 3)metabolite-mediated invasion.

Recent research has revealed the presence of distinct microbial communities within breast tissue, with significant differences in composition between invasive carcinoma, DCIS, benign lesions, and normal tissue (El Tekle and Garrett, 2023), suggesting a potential role for microbiota in the transition from DCIS to IDC, with potential main roles including disruption of epithelial barrier and basement membrane, promotion of immune escape, and metabolite-mediated invasion ((**Figure 3**). EMT serves as a critical indicator of epithelial barrier disruption. It has been reported that BFT can induce E-cadherin cleavage and actin cytoskeleton reorganization, suggesting that BFT may represent a potential mechanism contributing to DCIS progression (Parida et al., 2021). It has been demonstrated that *Fusobacterium nucleatum* upregulates MMP9 expression, leading to basement membrane degradation and potentially contributing to the malignant progression from DCIS to IDC (Parhi et al., 2020). Zhikai Mai et al. further demonstrated that S. multivorum enhanced the secretion of chemokines CCL20 and CXCL8 by tumor cells. The CCL20 secreted into the tumor microenvironment (TME) facilitated the recruitment of regulatory T (Treg) cells while concurrently reducing CD8+ T cell infiltration, thereby establishing an immunosuppressive microenvironment that promotes tumor immune escape through this mechanism (Mai et al., 2025). However, limited investigation exists regarding microbial-mediated immune escape during DCIS to IDC progression, warranting further exploration in this area. Furthermore, McCune et al. investigated the gut and oral microbiota across breast cancer patients, ductal carcinoma *in situ* (DCIS) patients, and healthy women, identifying that amino acid metabolism was significantly enriched within the oral microbiota of breast cancer patients relative to DCIS cohorts (Fu et al., 2022; McCune et al., 2024). This finding suggests that microbial-derived amino acid metabolism from oral sources may potentially contribute to DCIS progression. Currently, specific literature supporting the distinct microbial metabolic characteristics between DCIS and IDC remains limited. Overall, the precise mechanistic roles of microbiota in the DCIS progression process have not been systematically elucidated, representing a promising avenue for future investigation.

## 8 Microbiota-mediated regulation of breast cancer therapy and prognosis

Gut microbiota not only participates in the initiation, progression, and metastasis of breast cancer, but also be associated with patient prognosis, affecting responses to radiotherapy, chemotherapy, and immunotherapy (Guo, 2024).

Studies have shown that the abundance of *Pseudomonas* increases in breast tumor tissues following neoadjuvant chemotherapy, whereas *Prevotella* tends to decrease in tumors of untreated patients. In patients with distant metastases, the primary breast tumor exhibits significant enrichment of *Brevundimonas* and *Staphylococcus*. Gut microbial composition is also closely associated with postoperative chronic pain, therapeutic efficacy, and adverse effects in breast cancer patients. For instance, increased levels of certain commensal bacteria may correlate with chemotherapy-related side effects, negatively impacting prognosis. Irinotecan treatment itself can induce microbiota dysbiosis, exacerbating drug toxicity; meanwhile, enrichment of *Clostridium* and *Enterobacteriaceae* in the gut promotes β-glucuronidase production, potentially accelerating tumor progression. A probiotic combination formulation comprising Bifidobacterium breve and Bifidobacterium longum has been demonstrated to augment the therapeutic efficacy of anti-PD-L1 immunotherapy.

Against this backdrop, bacteria-targeted therapeutic strategies have emerged as a research focus. Experimental evidence indicates that Fusobacterium nucleatum–guided nanocarriers can deliver antibiotic-loaded liposomes selectively to tumor sites, eradicating intratumoral bacteria and substantially restoring chemosensitivity (Peng et al., 2024). Additionally, bacterial-derived extracellular vesicles have been shown to enhance immunochemotherapeutic efficacy in triple-negative breast cancer (TNBC) and ameliorate immunosuppressive conditions within the tumor microenvironment. Microbiota composition is also closely linked to the expression of immune-related genes and sensitivity to chemotherapeutic agents, such as tamoxifen and docetaxel, further highlighting its potential role in personalized breast cancer therapy.

Distinct molecular subtypes may exhibit unique microbiome characteristics, necessitating individualized assessment. Specifically, triple-negative breast cancer (TNBC) displays distinctive microbial signatures, with studies demonstrating that doxorubicin treatment increases the abundance of Akkermansia muciniphila, whereas high-fat diet-induced microbiota alterations diminish the therapeutic efficacy of doxorubicin (Bawaneh et al., 2022; Chen J. et al., 2024). The microbial metabolite trimethylamine N-oxide (TMAO) has been demonstrated to activate CD8+ T cell-mediated anti-tumor immunity in specific breast cancer subtypes (Wang et al., 2022). For HER2-positive breast cancer cells, sodium butyrate exhibits substantial anti-tumor activity either as monotherapy or in combination with trastuzumab. CD8+ T cells play a pivotal role in HER2/neu+ breast cancer pathogenesis, with TMAO produced by Clostridium species serving to potentiate this immunomodulatory effect (Mirji et al., 2022; Wang et al., 2022; Lu et al., 2024). Furthermore, differential microbial abundance profiles have been identified between aromatase inhibitor-resistant and sensitive patients in hormone receptor-positive (HR+) breast cancer (Lasagna et al., 2022). Longitudinal analysis of gut microbiota composition during endocrine therapy in HR+ patients revealed significant enrichment of Blautia following hormone therapy administration (Tsai et al., 2025). However, systematic characterization of microbial signatures across distinct molecular subtypes (including Luminal A, Luminal B, HER2+, and others) in breast cancer remains to be established. Future research needs to be stratified by molecular subtypes and treatment backgrounds, and subtype-specific microbial biomarkers need to be developed.

## 9 Conclusions and discussion

As essential components of the breast cancer ecosystem, microorganisms collectively modulate breast carcinogenesis through multiple mechanisms involving estrogen pathways, microbial metabolites-induced genotoxicity, and immunoregulatory-inflammatory interactions.

Given the specificity of microbial communities within breast tissue and their close association with tumor progression, future strategies may leverage microbiota for novel preventive, diagnosis, risk-stratification, and therapeutic interventions. A recent systematic review and meta-analysis has further quantified alterations in gut microbiota diversity among patients with breast cancer. Dysbiosis—characterized by reduced microbial diversity and impaired community stability—has been associated with an increased risk of tumorigenesis, thereby offering emerging evidence for its potential role in early detection (Gamba et al., 2025). In addition, accumulating studies suggest that, compared with healthy individuals, the overrepresentation of specific gut commensal bacteria in breast cancer patients may be linked to poorer clinical outcomes (Terrisse et al., 2021). Nevertheless, most of the available evidence is derived from cross-sectional studies, limiting causal inference and underscoring the need for further mechanistic and longitudinal investigations.

Translational applications are beginning to emerge: in therapy, probiotic formulations combining *Bifidobacterium breve* and *Bifidobacterium longum* can modulate microbial balance and exert antitumor effects; dietary interventions such as inulin supplementation can promote *Bifidobacterium* proliferation and increase butyrate levels, thereby inhibiting breast tumor growth; synthetic biology approaches to engineer bacteria as targeted drug delivery vectors also show promise.

With respect to endocrine therapy, researchers have focused on integrated analyses of gut microbiota and metabolomics among breast cancer patients undergoing adjuvant endocrine therapy (Sheikh et al., 2025), Lasagna et al. conducted an observational cohort study focusing on luminal breast cancer patients, examining the association between gut microbial diversity and treatment response in postmenopausal women receiving aromatase inhibitors. Their results demonstrated significant differences in the mean relative abundance of specific microbial taxa between patients with therapeutic resistance and those who remained sensitive to treatment, suggesting a potential link between gut microbiota composition and endocrine responsiveness (Lasagna et al., 2022), Additionally, studies have reported the effects of long-term endocrine therapy on gut microbial composition, revealing that patients experiencing recurrence exhibit diminished alpha diversity and elevated abundances of specific bacterial taxa (Hou et al., 2026). In the field of immunotherapy, CAR-T technology, which introduces chimeric antigen receptors into T cells to enable efficient tumor cell recognition and elimination, represents a significant breakthrough in cancer treatment. However, its application in solid tumors remains constrained by heterogeneity in target expression. To address this, Rosa L. Vincent and colleagues developed a multifunctional probiotic system capable of co-releasing chemokines, effectively enhancing CAR-T cell recruitment to tumor sites and improving therapeutic responses.

Although a growing body of research has identified associations between the microbiota and breast cancer, the overall strength and reliability of this evidence require careful scrutiny. Several methodological and conceptual limitations should be considered. First, the majority of existing human studies are cross-sectional in design, and thus inherently limited to identifying correlations rather than establishing causality. Second, technical constraints remain a significant concern. Most investigations rely on 16S rRNA gene sequencing, which typically provides taxonomic resolution only at the genus level, thereby limiting biological interpretation. While shotgun metagenomic sequencing offers improved resolution, its sensitivity may be compromised when detecting low-abundance microbial signals. This challenge is particularly pronounced in low-biomass tissues such as breast tumors, where contamination from reagents, laboratory environments, or adjacent tissues can disproportionately influence results, Eisenhofer et al. also put forward some suggestions regarding the contamination issue in studies of low-microbial-biomass microbiomes (Eisenhofer et al., 2019). Consequently, rigorous implementation of negative controls and independent validation strategies is essential to ensure data reliability. Third, reproducibility across cohort studies remains suboptimal, largely due to inter-individual variability and batch effects, which are especially problematic in low-biomass microbiota analyses. Fourth, many studies are constrained by relatively small sample sizes, limiting statistical power and complicating subtype-specific analyses in breast cancer. Finally, the heterogeneity in study design—including the integration of human, animal, and *in vitro* data—further complicates interpretation. Mechanistic insights are predominantly derived from animal models and *in vitro* systems, which may not fully recapitulate human physiological conditions and therefore require cautious extrapolation. Taken together, these limitations underscore the need for careful interpretation of current findings and highlight the necessity for more robust, well-controlled, and large-scale studies to clarify the role of the microbiota in breast cancer.

From a diagnostic perspective, triple-negative breast cancer (TNBC) has been shown to harbor unique microbial signatures, yet comprehensive microbiome profiles for other molecular subtypes, such as Luminal A, Luminal B, and HER2^+^ tumors, remain unestablished. Future work may focus on constructing diagnostic models based on microbial features in tissues or bodily fluids (blood, feces) and integrating multi-omics data to elucidate microbial mechanisms in breast cancer. Leveraging specific microbial signatures, alone or in combination with conventional imaging and molecular biomarkers, to assess tumor grading, staging, recurrence risk, and overall survival represents a promising direction for further investigation.
